# Teaching approaches and strategies that promote healthy eating in primary school children: a systematic review and meta-analysis

**DOI:** 10.1186/s12966-015-0182-8

**Published:** 2015-02-25

**Authors:** Dean A Dudley, Wayne G Cotton, Louisa R Peralta

**Affiliations:** School of Education, Faculty of Human Sciences, Macquarie University, Sydney, NSW Australia; Faculty of Education and Social Work, University of Sydney, Sydney, NSW Australia

**Keywords:** Elementary school, Nutrition, Fruit, Vegetable, Sugar, Energy intake, Knowledge, Systematic review

## Abstract

**Background:**

Healthy eating by primary school-aged children is important for good health and development. Schools can play an important role in the education and promotion of healthy eating among children. The aim of this review was to: 1) perform a systematic review of randomised controlled, quasi-experimental and cluster controlled trials examining the school-based teaching interventions that improve the eating habits of primary school children; and 2) perform a meta-analysis to determine the effect of those interventions.

**Methods:**

The systematic review was limited to four healthy eating outcomes: reduced food consumption or energy intake; increased fruit and vegetable consumption or preference; reduced sugar consumption or preference (not from whole fruit); increased nutritional knowledge. In March 2014, we searched seven electronic databases using predefined keywords for intervention studies that were conducted in primary schools which focused on the four healthy eating outcomes. Targeted internet searching using Google Scholar was also used. In excess of 200,000 possible citations were identified. Abstracts and full text of articles of potentially relevant papers were screened to determine eligibility. Data pertaining to teaching strategies that reported on healthy eating outcomes for primary school children was extracted from the 49 eligible papers.

**Results:**

Experiential learning strategies were associated with the largest effects across the reduced food consumption or energy intake; increased fruit and vegetable consumption or preference; and increased nutritional knowledge outcomes. Reducing sugar consumption and preference was most influenced by cross-curricular approaches embedded in the interventions.

**Conclusions:**

As with most educational interventions, most of the teaching strategies extracted from the intervention studies led to positive changes in primary school children’s healthy eating behaviours. However, given the finite resources, increased overcrowding of school curriculum and capacity of teachers in primary schools, a meta-analysis of this scope is able to provide stakeholders with the best evidence of where these resources should be focused.

## Introduction

### Rationale

The Australian National Health and Medical Research Council (2013) [1] states that optimum nutrition is essential for the healthy growth and development of children. Healthy eating contributes to achieving and maintaining a healthy weight, and provides protection against chronic disease and premature mortality. Conversely, unhealthy eating early in life, in particular the over-consumption of energy-dense, nutrient-poor foods and drinks, as well as physical inactivity and a sedentary lifestyle, are predictors of overweight and obesity [2,3]. There is good evidence that many other non-communicable diseases (such as diabetes, osteoporosis, and hypertension) are also related to unhealthy eating habits and patterns formed during childhood [4]. As such, it is important to establish healthy eating behaviours early, as evidence shows that eating habits and patterns track into adulthood [5,6]. Therefore, childhood is a period where education about healthy eating is essential for establishing healthy eating practices in later years. Schools have been a popular setting for the implementation of health promotion and prevention interventions, as they offer continuous, intensive contact with children and that lifelong health and wellbeing begins with promoting healthy behaviours early in life [7]. School infrastructure, physical environment, policies, curricula, teaching and learning, and staff all have the potential to positively influence child health. Whilst schools have remained a popular infrastructure for health promotion initiatives, teachers will remain the key agent of promoting health and nutrition within schools post-2015 [8]. No systematic review or meta-analysis have been undertaken to date which ascertain the strategies teachers should employ in order to yield maximum effect from their teaching interventions when it comes to fostering healthy eating behaviours in primary school-aged children.

### Objectives

Our aim was to systematically review the evidence related to interventions designed to improve healthy eating habits and patterns of primary school students. Our objectives were to: 1) describe the nature of the interventions that had been conducted (i.e., theories and teaching strategies and approaches); and 2) conduct meta-analyses to determine the effectiveness of these interventions.

## Methods

### Design

This systematic review and meta-analyses report on data extracted and synthesised in 2014 as part of a review project undertaken for the New South Wales (NSW) Department of Education and Communities and the NSW Ministry of Health. The PRISMA (Preferred Reporting Items for Systematic Review and Meta-Analysis) Statement [9] was followed to ensure the transparent reporting of the study.

### Eligibility criteria

#### Interventions types

We included teaching and school-based elementary school interventions delivered by teachers or teacher substitutes that sought to bring about positive nutritional consumption, preference or knowledge change in elementary school children. The following types of teaching and school-based interventions included:

1. Curriculum initiatives or evaluations
2. Nutrition-friendly school initiatives
3. Community programs linked to curricula or delivered by schools (e.g. community gardens)
4. Health/nutrition education programs related to improving dietary habits
5. Environmental school change strategies implemented by classroom teachers
6. Environmental interventions/industry partnerships focused on point-of-purchase consumption linked through classroom based education; this might include campaigns to draw attention to healthier products in school canteens or school lunch choices
7. Social marketing campaigns
8. Policies that seek to improve dietary habits of elementary school children (i.e. school board level, provincial/national level).

Acceptable designs for this review included randomised, quasi-experimental and cluster controlled studies conducted in elementary schools (Grades K-6) whereby the primary change agent in the intervention was the classroom teacher (or their teaching substitute). Relevant clusters within studies included individual students, classrooms, schools or communities as the unit of analysis.

#### Locations

Intervention locations had to include elementary schools and/or their immediate community settings. We excluded programs or strategies delivered solely through homes, religious institutions other than schools, non-governmental organisations, primary health care settings, universities, hospitals, outpatient clinics located within hospital settings, commercial programs and metabolic or weight loss clinics.

#### Outcomes of interest (Healthy eating behaviours)

Our primary outcomes included student consumption, preference and knowledge of nutrient dense foods. Evidence of intervention effects included measures at individual, family, school or community levels. They also included measures of food consumption, preference or knowledge and change in food environments, food disappearance, and food sales (in school cafeterias). Measures of consumption included: diet and food intake records, self-reported and/or reported by parents, teachers or both; food frequency questionnaires/balance sheets; food wastage and plate waste; and micronutrient measures (i.e., biomarkers of exposure to food). Measures of preference included: questionnaires, surveys or self-report instruments that included Likert scales, pairing activities, or self-reported preferences. Measures of knowledge included: questionnaires or tests on food-related knowledge (i.e., Recommended Dietary Intakes, ingredients, nutritional knowledge).

These primary outcomes were then grouped into four dominant healthy eating outcomes that the authors determined aligned with the National Health and Medical Research Council (NHMRC) and their Healthy Eating for Children [10] Guidelines. Our outcomes were therefore;

1. Food Consumption and Energy Intake–NHMRC Guideline 1 (Limiting energy intake to meet energy needs)
2. Fruit and Vegetable (FV) Consumption or Preference–NHMRC Guideline 2 (Enjoy a wide variety of nutritious foods)
3. Reduced Sugar Consumption or Preference (Not from whole fruit)–NHMRC Guideline 3 (Limit intake of foods containing added sugar)
4. Nutritional Knowledge–NHMRC Guideline 5 (Care for food)

Note: The instruments used and the number studies included in the review and meta-analysis did not allow for segregation of consumption and preference of fruit and vegetables or sugar. We acknowledge that preference for certain food types may have a greater affect on long-term consumption habits.

#### Outcomes of interest (Teaching strategies)

The primary outcomes of interest included any recognised teaching strategy or articulated approach to teaching that has a known effect on student learning and behaviour. The categorisation for these teaching strategies and approaches to curricula were largely derived from (but not limited to) those articulated in Hattie’s synthesis of meta-analysis relating to teaching, learning and student achievement [11].

### Search

Our search strategy included: electronic bibliographic databases; grey literature databases; reference lists of key articles; targeted internet searching via Google Scholar; and targeted internet searching of key organisation websites.

We searched the following databases, adapting search terms according to the requirements of individual databases in terms of subject heading terminology and syntax: PUBMED; MEDLINE; the Cochrane Central Register of Controlled Trials (CENTRAL); PsycINFO; ERIC; ScienceDirect; and A + Education. These search terms were based on; 1) participants (e.g. child* OR young people OR youth OR pediatric OR paediatric OR primary school-age* OR elementary school-age* OR primary student* OR elementary student* OR primary school* OR elementary school*); 2) delivery (e.g. teach* OR class* OR health* ed* teach* OR learn* OR teach* polic* OR nutrition ed* OR health* eat*); 3) strategies (e.g. phys* edu* OR health* edu* OR curricul* OR outdoor* OR cook* OR food* OR fruit* OR veg* OR know* OR test*); 4) design (e.g. RCT OR randomi* OR control* OR trial* OR evaluat* OR quasi-exper* OR cluster*). The dates range for search were from database inception to 31^st^ May, 2014.

The search results were then refined to include the full text copies retrieved from these databases and Google Scholar that were published after 1970. These citations were then cross-referenced electronically with 15 reference lists from scoping and systematic review papers in the field of nutrition, education, and health promotion published between 1997 and 2012. A final database and internet search was then conducted to identify studies published between January 2010 (year prior to publication of most recent systematic review) and May 2014.

### Screening of citations

Initially duplicate citations were removed from the search by the lead author. The abstract of each citation was then reviewed by a single researcher (DAD) to determine whether it would be included in the systematic review. The full-text articles of all potentially relevant citations were obtained and saved as Adobe-PDF files. Whenever it was uncertain as to whether a citation was appropriate, the full-text copy was obtained. The lead author then screened the citation list. Citations that were deemed ineligible were reviewed by the remaining two authors (WGC, LRP) to determine if any potentially relevant citations were missed, and full-text copies of these citations were then obtained.

### Study selection

Following the screening process, full-text articles were then reviewed by the three researchers against the inclusion criteria; if uncertain as whether or not to include an article, the article in question was reviewed again until a final decision was made by majority consensus.

### Data extraction

Data was initially extracted from the included studies by the lead researcher from full-text articles and placed in tabulated form (see Table 1). This data included:

**Table 1:** **Studies examining the teaching strategies/approaches used to promote healthy eating to primary school students**

**Author, Year, Country, Funding agency**	Design, Dominant/Theory Framework*	**Sample**	**Treatment Length**	**Teaching Strategy/Approach**	**Coupled with Physical Activity**	**Coupled with Specially-resourced teachers**	**Relevant Outcome Categories**	**Statistical Significance (p** ***value*** **/95% CI)**	**Effect Size (Cohen’s** ***d*** **)**		
									*M*_1_ - *M*_2_/SD_pooled_		
**Randomised Controlled Trials**											
Francis et al. (2010) Trinidad & Tobago, Self-funded [40]	RCT/NR	579 x Grade 6 students	32 weeks	(Curriculum approach) Bloom’s mastery learning model	√	X	Children’s Eating Attitude Test-26 (M)	<0.05	0.20		
		Mean age: 10.4 yrs					**Self Report**				
							SLB consumption (Servings/wk)	NS	−0.42		
							Fried food consumption (Servings/day)	0.04	−0.21		
							HFSS food consumption (<502 kJ/day)	NS	−0.21		
**Quasi-experimental Trials**											
Auld et al. (1998) USA, Kraft Foods [43]	QE/SCT, CDT	851 x Grades K-5 students	4 years	Cross-curricular & experiential learning	X	√	FV Consumption (Plate waste)	<.001	Insufficient data reported for calculation		
		Mean age: NR					**Self-efficacy (Likert scale)**				
							- Food prep	<.01			
							- Eating FV	<.01			
							**4th/5th Grade Knowledge (Test)**				
							- Food Pyramid	<.001			
							- Ingredients	<.001			
Bell & Lamb (1973) USA, Dairy Council Inc [44]	QE/NR	1913 x Grade 5 students	6 weeks	Popham Instruction Model (Define behavioural objectives, Diagnose student needs, Present learning opportunities, Evaluate attainment)	X	X	Milk consumption (oz.)	NS	Insufficient data reported for calculation		
							Vegetable consumption (oz.)	.05			
		Mean age: NR					Nutrition knowledge (Test)	.001			
Edwards & Hermann (2011) USA, NR [45]	QE/NR	11 x Grade 1 students	3 weeks	Literary abstraction	X	X	Legume taking (Number)	.05	Insufficient data reported for calculation		
		Mean age: NR					Legume tasting (Number)	.14			
Fahlman et al. (2008) USA, NR [46]	QE/NR	576 x students	4 weeks	(Curriculum approach) adapted Health Belief Model	X	√	**24 hr recall of Daily Dietary Intake**				
		Mean age: 12.2 yrs					- Grain consumption (Servings/day)	NS	−0.08		
							- Fruit consumption(Servings/day)	.047	0.97		
							- Vegetable consumption (Servings/day)	.018	0.49		
							- Dairy consumption (Servings/day)	NS	0.02		
							- Meat consumption (Servings/day)	NS	−0.02		
							**Self Efficacy (Likert scale)**				
							- Eat more FV	NS	1.11		
							- Eat less fat	NS	−0.15		
							- Drink less SLB	NS	−0.05		
							- Eat healthy at FF restaurants	NS	1.30		
Friel et al. (1999) Ireland, Dept of Health [47]	QE/SLT	821 x Grades 3–4 students	10 weeks	Cross-curricular	X	√	**Food Pairing Questionnaire**				
		Mean age: NR					- Behaviour	<.01	0.72		
							- Preference	<.01	1.00		
							- Knowledge	NS	−0.29		
Gortmaker et al. (1999) USA, Walton Family Foundation [48]	QE/SCT BCT	336 x Grades 4–5 students	2 years	Cross-curricular (Math, science, language, social studies, physical education) coupled with a Social Marketing Approach	√	X	**24 hr recall of Daily Dietary Intake**		Insufficient data reported for calculation		
		Mean age: 9.1 yrs					- Energy from fat (%)	.04			
							- FV consumption (Servings/4184 kJ)	.01			
							- Vitamin C (mg/4184 kJ)	.01			
Govula et al. (2007) USA, NR [30]	QE/NR	33 x Grade 3 students	6 weeks	(Curriculum approach) MyPyramid and Medicine Wheel Nutrition for Native Americans	X	X	**Block Kids Fruit/Vegetable recall**				
							- F&V consumption (Servings/per day)	.010	.10		
		Mean age: NR		Culturally appropriate lessons			- Fruit consumption (Servings/per day)	.519	−0.26		
							- Vegetable consumption (Servings/per day)	<.001	1.04		
							Knowledge Questionnaire (% correct)	<.001	Insufficient data		
Horne et al. (2004) UK, Horticultural Development Council, Fresh Produce Consortium, ASDA, Co-operative Group, Safeway, Sainsbury, Somerfield, Tesco, Bird’s Eye [31]	QE/SLT	749 x Grades K-6 students	16 weeks	Animation abstraction and contingent reinforcement for F&V consumption	X	X	**Consumption based on teacher visual estimates**				
		Mean age: NR					- 5-7 yr/old fruit (%)	<.002	2.12		
							- 5-7 yr/old vegetable (%)	NR	2.01		
							- 7-11 yr/old fruit (%)	<.002	2.36		
							- 7-11 yr/old vegetable (%)	NR	1.51		
Liquori et al. (1998) USA, NR [49]	QE/SCT	590 x Grades K-6 students	1 year	Experiential learning (Cooking, environment and community garden)	X	√	Food intake based on teacher visual estimates (%)	**Grd K-3**	**Grd 4–6**	**Grd K-3**	**Grd 4-6**
								<.01	NS	−1.90	−2.03
		Mean age: NR					**Self report**				
							- Preference for plant food	<.001	<.001	2.51	0.00
							- Attitudes	NS	NS	0.59	0.04
							- Knowledge	<.05	<.001	1.98	1.94
							- Self efficacy in cooking	NS	<.05	0.79	0.70
							- Food intentions	<.01	NS	0.63	−0.17
							- Paired food choice	<.01	NS	1.58	−0.06
Manios et al. (2002), Greece, Kellogg’s, Greek Ministry of Sport, Greek Ministry of Education [50]	QE/NR	1006 x Grade 1 students	6 years	(Curriculum approach) Literary abstraction	√	√	**Parental reporting (Food Diary)**				
		Age range: 5.5-6.5 yrs					- Energy (kJ)	<.05	−0.38		
							- Total fat (g)	<.05	−0.38		
							- Protein (g)	<.05	−0.42		
							- Carbohydrate (g)	NS	−0.23		
McAleese & Rankin (2007), USA, NR [36]	QE/NR	99 x Grade 6 students	12 weeks	(Curriculum approach) *Nutrition in the Garden*	X	X	**24 hr recall of Daily Dietary Intake**				
		Mean age: 11.11 yrs		Experiential learning (School garden)			- Fruit (Servings/day)	<.001	1.17		
							- Vegetables (Servings/day)	<.001	0.92		
							- Vitamin A (μg/day)	.004	0.20		
							- Vitamin C (mg/day)	.016	0.49		
							- Fibre (g/day)	.001	0.56		
Morgan et al. (2010) Australia, Hunter Medical Research, Coles [51]	QE/SCT	127 x Grades 5–6 students	10 weeks	(Curriculum approach) Nutrition in the Garden – Modified	X	X	FV knowledge (Gimme 5 Questionnaire)	<.02	Insufficient data reported for calculation		
		Age range: 11-12 yrs		Experiential learning (School garden)			**24 hr recall of Daily Dietary Intake**	.22			
							- Vegetable intake (Servings/day)	.23			
							- Fruit intake (Servings/day)				
Simmons-Morton et al. (1991), USA, HHLBI Grant [52]	QE/SCT	135 x Grades K-4 students)	40 weeks	(Curriculum approach) Behaviour-based Health & Physical Education	√	√	**24 hr recall of Daily Dietary Intake**				
		Mean age: NR		(Canteen) New School Lunch			- Analysis of tray lunch (% kcals)	<.05	−0.10		
							- Analysis of bag lunch (% kcals)	<.05	0.03		
**Cluster-Controlled Trials**											
Agozzino et al. (2007), Italy, NR [53]	CT/CogT	570 x students (30 x 4 & 5 grade classes)	40 weeks	(Curriculum approach) Didactic-approach to health education	X	√	**24 hr recall of Daily Dietary Intake**		Insufficient data reported for calculation		
							- Breakfast consumption (Sufficient)	<.001			
							- Meat consumption (Sufficient)	.003			
							- Fish consumption (Sufficient)	.02			
							- Pulse consumption (Sufficient)	.003			
							- Vegetable consumption (Sufficient)	<.001			
Amaro et al. (2005), Italy, Amici di Raoul Follereau (AIFO) [54]	CT/NR	241 x students Mean age: 12.4 yrs	24 weeks	Kalèdo Board Game (15-30mins play time p/w)	X	X	Nutritional knowledge (31 items)	<0.05	Insufficient data reported for calculation		
							BMI (z-score)	NS			
Anderson et al. (2005), UK, Food Standards Agency [55]	CT/TPB	129 x Grades 1–6 students	36 weeks	(Curriculum approach) based on experiential learning, video & literary abstraction	X	X	**Cognitive & attitudinal (Likert scale)**				
		Mean age: 8.5 yrs		Marketing and canteen provisions			- Diet heart disease knowledge	.001	0.24		
							- Preference for HFSS foods	.034	−0.32		
							**3-day food diary**				
							- FV consumption (g)	.617	0.07		
							- Energy (kJ)	.327	0.00		
							- Sucrose (g)	.578	0.01		
Baronowski et al. (2000), USA, NR [32]	CT/SCT	3347 x Grades 4–6 students	12 weeks	(Curriculum approach) Gimme 5	X	X	**7-day food record**				
		Mean age: NR		Experiential learning, goal setting & problem solving, contingent reinforcement for F&V consumption			- FV consumption (Servings)	<.05	0.03		
							- Vegetable consumption(Servings)	<.01	0.00		
							**Questionnaire (Likert scale)**				
							- Self efficacy (Eating FV)	<.10	0.02		
							- Social norms	<.10	0.00		
							- Asking behaviour	<.05	0.06		
							- Knowledge	<.05	0.05		
Bere et al. (2006), Norway, Norwegian Research Council [56]	CT/SCT	369 x Grade 6 students	28 weeks	(Curriculum approach) National Curriculum	X	√	**24 hr recall of Daily Dietary Intake**		Insufficient data reported for calculation		
		Mean age: 11.3 yrs		Experiential learning (Cooking/Food Prep)			- FV consumption (Servings per day)	.41			
							Curriculum enjoyment (Likert scale)	.004			
Cooke (2011), UK, Medical Research Council National Prevention Research Initiative [57]	CT/mixed	442 x Kindergarten students	2 weeks	Contingent reinforcement for vegetable tasting	X	X	Liking of vegetables (Likert scale)	.001	Insufficient data reported for calculation		
		Mean age: 6 yrs					Intake of vegetables	.001			
Day et al. (2008), Canada, NR [58]	CT/NR	444 x Grades 4–5 students	12 weeks	Integrates classroom learning, environmental change strategies, and a family/community component to promote the consumption of FV	X	X	**24 hr recall of Daily Dietary Intake**				
		Mean age: 10.0 yrs					- Fruit consumption (Servings)	<.05	−0.04		
							- Vegetable consumption (Servings)	NS	−0.05		
							- F V consumption (Servings)	<.05	−0.06		
							- Variety of FV consumption (Servings)	<.05	−0.01		
Domel et al. (1993) USA, The International Apple Institute [59]	CT/SCT	301 x Grades 4–5 students	6 weeks	“5 a Day - for Better Health”	X	X	**24 hr recall of Daily Dietary Intake**				
							- F V consumption (Servings)	NS	0.47		
							- Fruit consumption (Servings)	.001	0.74		
							- Juice consumption (Servings)	NS	−0.14		
							- Vegetable consumption (Servings)	.018	0.28		
							- Legume consumption (Servings)	NS	0.45		
							**Questionnaire (Likert scale)**				
							- Fruit	.046	0.35		
							- Vegetables	NS	0.32		
							FV Knowledge (Multiple choice score)	<.001	0.59		
Duncan et al. (2011) New Zealand, Health Research Council NZ [60]	CT/NR	97 x Grades 5–6 students	6 weeks	Curriculum approach with “Healthy Homework” Teaching Resource	√	X	**Food Diaries**		Insufficient data reported for calculation		
		Mean age: NR		Experiential learning (Cooking)			- Fruit consumption (Servings/per day)	NS			
							- Vegetable consumption (Servings/per day)	.016			
							- Unhealthy food consumption (Servings per/day)	.042			
							- Unhealthy drink consumption (Servings/per day)	NS			
Foster et al. (2008), USA, CDC, US Department of Agriculture/Food and Nutrition Service [61]	CT/NR	1349 x Grades 4–6 students	2 years	The School Nutrition Policy Initiative included the following components: school self-assessment, nutrition education, nutrition policy, social marketing, and parent outreach. Cross-curricular/Integrated learning	√	X	BMI (z score)	.80	Insufficient data reported for calculation		
		Mean age: 11.2 yrs					Total Energy (kJ/day)	.12			
							Total Fat (g/day)	.12			
							FV Consumption (Servings/per day)	.82			
Gorely et al. (2009) UK, Great Run, Coca-Cola Company [62]	CT/SCT	589 x students	40 weeks	(Curriculum approach) Physical education lessons and homework tasks	√	X	**24 hr recall of Daily Dietary Intake**		Insufficient data reported for calculation		
		Mean age: 8.8 yrs		Fun run event			- FV Consumption (Servings/per day)	NS			
							Knowledge of healthy lifestyle (MC Test)	NS			
Head (1974) USA, Emergency food and Medical Services [63]	CT/NR	4,700 x Grades 5, 7 & 10 students	20 weeks	Cross Curriculum approach in nutrition, reading, math, history, art, music and science	X	X	Knowledge (% correct)	<.05	Insufficient data reported for calculation		
		Mean age: NR					School lunch (% of plate waste)	<.05			
							Acceptance of school served food (%)	NS			
Hendy et al. (2011) USA, grants from Penn State University [64]	CT/SCT	382 x Grades 1–4 students	12 weeks	Kid’s Choice Program (KCP), contingent reinforcement supported by parental involvement.	√	X	Eating FV first in meals	<.001	Insufficient data reported for calculation		
	SDT						Choosing low fat and low sugar drinks	<.001			
Hoffman et al. (2010) USA, National Institute of Child Health and Human Development [65]	CT/SLT	297 x Kindergarten & Grade 1 students	2 years	Cross-Curricular program included school-wide, classroom, lunchroom, and family components	X	X	**Plate Waste Weight**	Year 1	Year 2	Year 1	Year 2
							- Fruit intake(g)	<.001	<.001	0.86	0.55 -
							- Vegetable intake (g)	<.01	NS	0.34	
James et al. (2005) UK, GlaxoSmithKline, Aventis, Pfizer, Florence Nightingale Foundation [33]	CT/NR	644 x 2nd-6th Grade students	40 weeks	(Curriculum approach) Reducing SLB consumption	X	√	**3-Day Consumption Diary**				
		Mean age: 8.7 yrs		Cross curricular approach in Health, Science, Music and Art			- SLB Consumption (Servings)	0.02	0.83		
Kipping (2010), UK, Department of Health [66]	CT/SCT	393 x Grade 5 students	20 weeks	(Curriculum approach) Eat Well Keep Moving program	√	X	**24 hr recall of Daily Dietary Intake**		Insufficient data reported for calculation		
	BCT	Mean age: 9.4 yrs					- FV consumption (Servings/per day)	NS			
							- Snack consumption (Servings/per day)	NS			
							- HFF consumption (Servings/per day)	NS			
							- SLB consumption (Servings/per day)	NS			
Kristjansdottir et al. (2010) Iceland, The University of Iceland, The Icelandic Centre for Research, Brim Seafood [67]	CT/NR	171 x Grade 2 students	2 years	(Curriculum approach) co developed with teachers and supported by homework, letters to parents and meetings with parents	X	X	**Food record by parents**				
		Mean age: NR					- FV consumption (g/day)	<.001	0.93		
							- Fruit consumption (g/day)	.001	0.62		
							- Vegetable consumption (g/day)	<.001	1.35		
Luepker et al. (1996) USA, National Heart, Lung, and Blood Institute [68]	CT/NR	5106 x Grade 3 students	3 years	(Curriculum Approach) (Child and Adolescent Trial for Cardiovascular Health-CATCH)	√	X	**School Lunch Menu Analysis**				
		Mean age: NR		Enhanced PE and classroom health curricula. 28 additional schools received these components plus family education.			- Total energy intake (MJ)	<.001	0.02		
							**24 hr recall of Daily Dietary Intake**				
							- Total energy intake (MJ)	.01	0.07		
							- Total energy from fat (%)	.001	0.17		
							**Health Behaviour Questionnaire**				
							- Dietary knowledge	<.001	0.25		
Mangunkusumo et al. (2007) The Netherlands, Organisation for Health Research and Development [69]	CT/NR	675 x 7th Grade students	12 weeks	Internet-tailored advice followed by dietary counselling	X	√	**24 hr recall of Daily Dietary Intake**		Insufficient data reported for calculation		
		Mean age: 10.3 yrs					-Vegetable consumption (g/per day)	NS			
							- Behavioural determinants	NS			
Muth (2008) USA, American Medical Association [70]	CT/SCT	73 x 4th Grade students	12 weeks	(Curriculum approach) (Improving Meals and Physical Activity in Children and Teens (IMPACT)	X	X	**Texas School Physical Activity and Nutrition Questionnaire (SPAN)**		Insufficient data reported for calculation		
		Mean age: 9.9 yrs		Train-the-trainer model with HS students trained to teach 4th graders			- FV Consumption (Servings/per day)	.05			
							- Nutritional knowledge (%)	.01			
Panunzio et al. (2007) Italy, NR [71]	CT/NR	471 x 4th Grade students	36 weeks	(Curriculum approach) Teachers vs Nutritionists	X	√	**24 hr recall of Daily Dietary Intake**		Insufficient data reported for calculation		
		Mean age: 9.6 yrs					- FV consumption (>1 serving p/day)	<0.01			
							- Legume consumption (>1 serving p/day)	<0.01			
							- Chips consumption (>1 serving p/day)	<0.01			
							- SLB consumption (>1 serving p/day)	<0.01			
Parcel et al. (1989) USA, National Heart, Lung, and Blood Institute [72]	CT/SLT	398 x K-4th Grade students	14 weeks	(Curriculum approach) 3 concurrent programs: the New School Lunch, Children’s Active Physical Education (CAPE), and Go For Health classroom instruction.	√	X	**Behavioural Capability Questionnaire**				
		Mean age: NR					- Diet behavioural capability (Score)	<.01	0.89		
							- Diet self-efficacy (Score)	NS	0.15		
							- Diet behavioural expectations (Score)	<.01	0.73		
							**Self-Reported Behaviour**				
							- Salt use (Daily use)	NS	0.00		
							- FV consumption (% of total intake)	NS	0.13		
Parmer (2009) USA, NR [73]	CT/ELT	115 x 2nd Grade students	28 weeks	(Curriculum approach)	X	X	**FV Survey (Likert Scale)**				
		Mean age: 7.3 yrs		Nutrition lessons + school garden			- MyPyramid food groups	NS	0.59		
				Experiential Learning (Gardening + Food Prep)			- Nutrient–food association	< .001	1.13		
							- Nutrient–job association	< .001	0.99		
							- F V identification	< .01	2.03		
							**Researcher Observed Lunch Choices**				
							- Vegetable choice (Servings)	<.01	1.09		
							- Vegetable consumption (Servings)	<.01	1.41		
Perry et al. (1998) USA, National Cancer Institute [74]	CT/SLT	441 x 4- 5th Grade students	40 weeks	The 5-a-Day Power Plus Program	X	X	**Researcher Observed Lunch Choices**		Insufficient data reported for calculation		
		Mean age: NR		- behavioural curricula			- FV consumption (Servings)	<.001			
				- parental involvement/education			- Vitamin A (μg)	.02			
				- school food service changes			- Vitamin C (mg)	<.001			
				- industry involvement and support.			**24 hr recall of Daily Dietary Intake**				
							- Fruit consumption (Servings)	.02			
							- Total fat consumption (%/kcal)	.02			
							- Calcium (mg)	.04			
							**Health Behaviour Questionnaire**				
							- Asking for F V (LIkert Scale)	.03			
							- Servings of FV (Nominal Scale)	<.001			
							- Knowledge of servings (Nominal Scale)	<.001			
Perry, Mulis et al. (1985), USA, NR [75]	CT/SLT	371 x 3rd-4th Grade students	10 weeks	(Curriculum approach) Hearty Heart and Friends program	X	X	**24 hr recall of Daily Dietary Intake**		Insufficient data reported for calculation		
	PBT	Mean age: NR					- Sugared cereal consumption (Less)	<.05			
							- Green vegetable consumption	<.02			
							- Fruit consumption	<.01			
							- Fried food consumption (Less)	<.005			
							- Added salt consumption (Less)	<.05			
Powers et al. (2005), USA. State Cooperative Extension System and State Department of Human Resources [76]	CT/SCT	1100 x 2nd- 3rd Grade students	6 weeks	Pizza Please Board Game with Nutrition education	X	√	Dietary Consumption Behaviour (Self report frequency)	<.001	0.23		
		Mean age: 7.6 yrs					- Dairy consumption	.001	0.22		
							- FV consumption	.016	0.15		
							Nutrition Knowledge (Item matching)	<.001	0.77		
							- Food appropriate Food Guide Pyramid	<.001	0.31		
							- Nutrient-food association	<.001	0.54		
							- Nutrient-job association	<.001	0.60		
Quinn et al. (2003) USA, Kappa Omicron Nu, Food Bank of Central New York [77]	CT/NR	126 x 5th Grade students	40 weeks	Experiential learning (Cooking) Modified CookShop program. Taught in schools with the support of parents	X	X	**24 hr recall of Daily Dietary Intake & Food Frequency Questionnaire (NCI)**				
							- Dietary Fibre (mg)	<.05	0.33		
							- Folate (mcg)	<.05	0.16		
							- Fruit consumption (Servings)	<.05	0.28		
							- Milk consumption (Servings)	<.001	0.47		
Resnicow et al. (1998) USA, National Heart, Lung and Blood Institute [78]	CT/SCT	966 x 4th - 5th Grade students	6 weeks	(Curriculum approach) Gimme-5 curriculum.	X	√	- FV preference (Likert scale)	<.001	Insufficient data reported for calculation		
		Mean age: NR		Teachers received the teacher wellness program involving 54 workshops over 2 yrs				(in favour of control)			
Reynolds et al. (2000) USA, National Cancer Institute Grant [79]	CT/SCT	1698 x 4th Grade students	2 years	High 5 intervention on FV consumption based around 3 interventions: classroom component, Parent component, Food Service component.	X	√	**24 hr recall of Daily Dietary Intake**		Insufficient data reported for calculation		
		Mean age:8.7 yrs					**-** Fruit consumption (Servings)	<.001			
							- Vegetables consumption (Servings)	<.001			
							- FV consumption (Servings)	<.001			
							- Calories from fat (%)	<.041			
							- Calories from carbohydrates (%)	<.017			
							- Fibre (g)	<.012			
							- Folate (μg)	<.034			
							- β-Catotine (μg)	<.034			
							- Vitamin C (mg)	<.048			
Sahota et al. (2001) UK, Northern and Yorkshire Region Research and Development Unit [80]	CT/NR	636 x 4th-5th Grade students Mean age: 8.4 yrs	40 weeks	Active programme promoting lifestyle in schools (APPLES program)	√	X	**24 hr recall of Daily Dietary Intake**		Insufficient data reported for calculation		
				Multidisciplinary, multiagency programme designed to influence diet and physical activity			- Vegetable consumption	<.05			
				Cross Curricular whole school community including parents, teachers, and catering staff							
Shannon & Chen (1988), USA, Pennsylvania State Department of Education [81]	CT/NR	1707 x 3rd Grade students	3 years	(Curriculum approach) *Nutrition in a Changing World (K-12 program).*	X	X	Knowledge (Test scores)	<.001	Insufficient data reported for calculation		
		Mean age:NR					Food attitudes (Likert scale)	<.001			
							Eating behaviours (24 hr recall)	<.001			
Smolak et al. (1998), USA, Ohio Dept of Education[82]	CT/NR	253 x 5th Grade students	24 weeks	(Curriculum approach) *Eating Smart, Eating for Me*	√	X	**24 hr recall of Daily Dietary Intake**		Boys	Girls	
		Mean age: NR					- Vegetable consumption (Servings)	NS			
								(<.05 by sex)	−0.31	0.24	
Spiegel & Foulk (2006), USA, Institute for America’s Health [83]	CT/TRA	1013 x 4th-5th Grade students	24 weeks	Wellness, Academics & You (WAY) Program	√	X	BMI (kg/m^2^)	0.01	−0.38		
		Mean age: NR		Cross-curricular – Language arts, mathematics, science & health education			FV Consumption (Survey)	NS	-		
Taylor et al. (2007), New Zealand, NR[34]	CT/NR	730 x primary students	2 years	APPLE Project - Community driven healthy eating & physical activity initiative.	√	√	**Three-day recall intakes**				
				Cross curricular school-based science nutrition lessons, recess activities & GoTri card game			**-** SLB consumption (Servings)	0.04	−0.21		
							- Juice consumption (Servings)	0.03	−0.24		
							- Water consumption (Servings)	0.07	0.24		
							- Fruit consumption (Servings)	<0.01	0.32		
							BMI (z-score)	<0.05	−0.49		
te Velde et al. (2007), The Netherlands, Norway, Spain, Commission of European Communities (RTD) programme[84]	CT/SCT	1472 x 5th-6th Grade students	52 weeks	Pro-children intervention (Three countries)	X	√	**24 hr recall of Daily Dietary Intake**				
		Mean age: 10.7 yrs		- Curriculum approach (w/web based feedback tool)			- FV consumption (g/d) All Countries	<0.02	0.18		
				- Free FV provision in schools			- FV consumption (g/d) Norway	<0.05	0.37		
				- Family web based feedback tool			- FV consumption (g/d) Spain	<0.05	0.15		
							- FV consumption (g/d) The Netherlands	<0.05	0.12		

TPB = Theory of Planned Behaviour; SCT = Social Cognitive Theory; CDT = Cognitive Development Theory; SLT = Social Learning Theory; BCT = Behavioural Choice Theory; CogT = Cognitive Theory; SDT = Self Determination Theory; GST = Group Socialization Theory; ELP = Experiential Learning Theory; PBT = Problem Behaviour Theory; TRA = Theory of Reasoned Action; RCT = Randomised controlled trial; QE = Quasi-experimental; CT = Cluster-controlled trial; NR = Not reported; NS = Not significant; FV = Fruit and vegetable; SLB = Sugar-laden beverages; HFSS = High fat, sugar & salt; HFF = High Fat Food; FF = Fast food, BMI = Body Mass Index.

1. Study authors;
2. Year of publication;
3. Country (s) of study;
4. Funding agency of study;
5. Study design;
6. Dominant Theoretical Framework used to inform study design
7. Study sample (Size, Grade, Mean age of participants);
8. Intervention length;
9. Whether the intervention was coupled with a physical activity or specially resourced teacher;
10. Relevant outcome categories
11. Statistical significance (p value/95%CI)
12. The effect size of different teaching strategies on each outcome (Cohen’s d). Note: If these were not reported in the study and Mean and Standard Deviations could be extracted either directly or indirectly, the Cohen’s d was calculated by the lead researcher and verified by the co-authors.

These data were tabulated by the lead author and shared with co-authors for feedback and review. Changes to these interpretations were decided by majority consensus by all three researchers.

The three researchers then reviewed each of the articles independently and each identified the teaching approaches employed in the intervention phase of the studies. Researchers met and cross-referenced their identification of each teaching approach and decided though consensus how each approach would be classified as a wider teaching strategy (if appropriate) that would allow for comparison between studies.

### Assessment of methodological quality

Included articles were also assessed for methodological quality using a 10-item quality assessment scale derived from van Sluijs and colleagues [12] (see Table 2). For each included article, three reviewers independently assessed whether the assessed item was present or if the assessed item was absent. Where an item was insufficiently described it was allocated an absent score. Agreement between reviewers for each article was set a priori at 80% [12]. That is, for each article, reviewers were required to agree that the items were either present or absent for 8 of the 10 items. In the case of less than 80% agreement, consensus was reached by further discussion. Results for the assessment of methodological quality are reported in Table 3.

**Table 2:** **Methodological quality assessment items (Adapted from van Sluijs et al. 2007)** [12 **]**

**Item**	**Description**
A	Key baseline characteristics are presented separately for treatment groups (age, and one relevant outcome (food consumption/energy intake; fruit and vegetable consumption or preference; reduced sugar consumption or preference; nutritional knowledge) and for randomised controlled trials and controlled trials, positive if baseline outcomes were statistically tested and results of tests were provided.
B	Randomisation procedure clearly and explicitly described and adequately carried out (generation of allocation sequence, allocation concealment and implementation)
C	Validated measures of food consumption/energy intake and/or fruit and vegetable consumption or preference and/or reduced sugar consumption or preference and/or nutritional knowledge (validation in same age group reported and/or cited)
D	Drop out reported and ≤20% for <6-month follow-up or ≤30% for ≥6-month follow-up
E	Blinded outcome variable assessments
F	Food consumption/energy intake and/or fruit and vegetable consumption or preference and/or reduced sugar consumption or preference and/or nutritional knowledge assessed a minimum of 6 months after pre-test
G	Intention to treat analysis for food consumption/energy intake and/or fruit and vegetable consumption or preference and/or reduced sugar consumption or preference and/or nutritional knowledge outcomes(s) (participants analysed in group they were originally allocated to, and participants not excluded from analyses because of non-compliance to treatment or because of some missing data)
H	Potential confounders accounted for in outcome analysis (e.g. baseline score, group/cluster, age)
I	Summary results for each group + treatment effect (difference between groups) + its precision (e.g. 95% confidence interval)
J	Power calculation reported, and the study was adequately powered to detect hypothesized relationships

**Table 3:** **Methodological quality and risk of bias assessment**

**Paper author/year**	**Assessment items**										
	**A**	**B**	**C**	**D**	**E**	**F**	**G**	**H**	**I**	**J**	**No. of criteria met**
	**Randomised controlled trials**										
Francis et al. (2010) [40]	√	√	√	x	x	√	x	√	√	√	7
	**Quasi-experimental trials**										
Auld et al. (1998) [43]	√	x	√	x	x	√	x	√	√	x	5
Bell & Lamb (1973) [44]	√	x	√	x	√	x	x	√	√	x	5
Edwards & Hermann (2011) [45]	√	x	√	x	x	x	x	x	√	x	3
Fahlman et al. (2008) [46]	√	x	√	x	x	x	x	x	x	x	2
Friel et al. (1999) [47]	√	x	√	√	x	x	x	x	√	x	4
Gortmaker et al. (1999) [48]	√	√	√	x	x	√	√	x	√	x	6
Govula et al. (2007) [30]	√	x	√	x	x	x	x	x	√	x	3
Horne et al. (2004) [31]	√	x	√	x	x	x	x	x	√	x	3
Liquori et al. (1998) USA, NR [49]	x	x	√	x	x	√	x	x	x	x	2
Manios et al. (2002) [50]	√	x	√	√	x	√	x	√	√	x	6
McAleese & Rankin (2007) [36]	√	x	x	x	x	x	x	x	√	x	2
Morgan et al. (2010) [51]	√	x	√	√	x	√	x	√	√	√	7
Simmons-Morton et al. (1991) [52]	√	x	x	x	x	√	x	x	x	x	2
	**Cluster-controlled trials**										
Agozzino et al. (2007) [53]	x	x	x	x	x	√	x	x	√	x	2
Amaro et al. (2005) [54]	√	x	x	√	x	√	x	√	√	√	6
Anderson et al. (2005) [55]	√	x	x	x	x	√	x	x	√	x	3
Baronowski et al. (2000) [32]	√	x	√	√	x	√	x	√	√	√	7
Bere et al. (2006) [56]	√	x	√	x	x	√	x	√	√	x	5
Cooke (2011) [57]	√	x	x	√	x	x	x	x	√	√	4
Day et al. (2008) [58]	√	x	√	√	x	x	x	√	√	x	5
Domel et al. (1993) [59]	√	x	√	√	x	x	x	√	√	x	5
Duncan et al. (2011) [60]	√	x	√	√	x	x	x	√	√	x	5
Foster et al. (2008) [61]	√	x	√	x	x	√	x	√	√	x	5
Gorely et al. (2009) [62]	√	x	√	√	x	√	√	√	√	x	7
Head (1974) [63]	√	x	x	x	x	x	x	x	x	x	1
Hendy et al. [64]	√	x	√	√	x	√	x	x	√	x	5
Hoffman et al. (2010) [65]	√	x	√	√	x	√	x	√	√	x	6
James et al. (2005) [33]	√	√	√	√	x	√	x	√	√	√	8
Kipping (2010) [66]	√	√	√	√	x	√	√	√	√	√	9
Kristjansdottir et al. (2010) [67]	√	x	x	√	x	√	x	√	√	x	5
Luepker et al. (1996) [68]	√	x	√	√	x	√	√	√	√	x	7
Mangunkusumo et al. (2007) [69]	√	x	√	√	x	√	√	√	√	√	8
Muth (2008) [70]	√	x	√	√	x	x	√	√	√	√	7
Panunzio et al. (2007) [71]	√	x	x	x	x	√	x	√	√	√	5
Parcel et al. (1989) [72]	√	x	x	√	x	√	x	√	√	x	5
Parmer (2009) [73]	√	x	x	x	x	√	x	√	√	x	4
Perry et al. (1998) [74]	x	x	√	√	x	√	x	√	√	x	5
Perry et al. (1985) [75]	x	x	√	x	x	x	x	√	√	x	3
Powers et al. (2005) [76]	x	x	√	x	x	x	x	x	√	x	2
Quinn et al. (2003) [77]	√	x	√	x	x	x	x	x	√	x	3
Resnicow et al. (1998) [78]	x	x	√	x	x	√	x	x	√	x	3
Reynolds et al. (2000) [79]	√	x	√	√	x	√	x	√	√	x	6
Sahota et al. (2001) [80]	√	√	x	√	x	√	x	x	√	√	6
Shannon & Chen (1988) [81]	x	x	√	√	x	√	x	√	x	x	4
Smolak et al. (1998) [82]	√	x	x	√	x	x	x	√	√	x	4
Spiegel & Foulk (2006) [83]	√	x	√	x	x	√	x	x	√	x	4
Taylor et al. (2007) [34]	√	x	√	x	x	√	x	√	√	√	6
te Velde et al. (2007) [84]	√	x	√	√	x	x	√	√	√	x	6

√ = criteria met; x = criteria not met.

### Synthesis of results

Effect sizes are the preferred metric for estimating the magnitude of effect of an intervention because they make possible between study as well as within study comparisons [13]. Cohen’s d, the effect-size metric constituting the focus of this meta-analysis, is one of the most widely used measures of magnitude of effect and commonly used in educational meta-analyses [11]. The formula for calculating Cohen’s *d* is:

$$ d=\left({M}_1-{M}_2\right)/S{D}_p, $$

where *M*_*1*_ is the mean of one group of study participants, *M*_*2*_ is the mean of a second group of study participants, and *SD*_*P*_ is the pooled standard deviation for both groups of study participants.

In instances where the groups have been given different learning experiences (e.g. an intervention), *d* is a measure of the magnitude of effect of the experience on the group receiving the enhanced teaching and learning experience. In cases where *SD* was not reported but *SE* (Standard Error) was, *SE* was converted to *SD* using the following formula:

$$ SD=SE\ \mathrm{x}\ \sqrt{\mathrm{N}} $$

As Cohen’s *d* accounts for sample size, mean effect sizes for the purposes of the meta-analysis were calculated as follows:

$$ {M}_d={\displaystyle \sum d/{\mathrm{N}}_{\mathrm{s}}} $$

where

*M*_*d*_ is the mean Cohen’s *d* calculated by the sum of all *d* values and divided by the number of studies (N_*s*_) from which a *d* value could be extracted for that outcome.

Data pertaining to each study were initially collated and described in a narrative summary (see Table 1). To facilitate comparison between the effect of teaching strategies/approaches, studies were divided according to their outcome measure as follows: Decreased food consumption/energy intake, increased FV consumption/preference, decreased sugar consumption/preference, and increased nutritional knowledge. Meta-analyses were conducted using the standardised mean difference approach (Cohen’s d) regardless of their statistical significance where at least two studies existed for a particular outcome measure and sufficient statistical data were reported to allow such synthesis to occur.

Studies incorporated into the meta-analyses included a comparison between teaching strategies/approaches and reported post-test/follow-up values or change scores along with measures of distribution (i.e. mean and standard deviation). For studies that included post-test and follow-up assessments, the assessments completed at the end of the study period (i.e., follow-up) were included in the meta-analyses. The standardized effect sizes were interpreted as minimal (<.02), small (0.2), medium (0.5), and large (0.8) [14]. Analyses also considered whether they represented an effective investment in education given the average effect size of most educational interventions is *d* = 0.4 [11].

## Results

### Study selection

**Figure 1:**
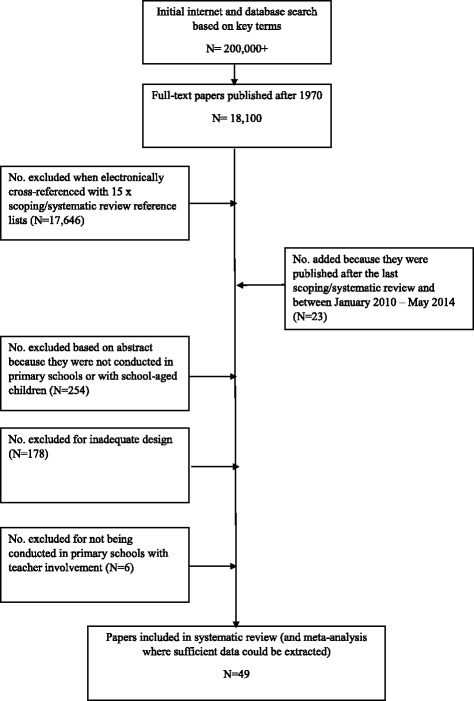
**Flowchart of study selection.**

The study selection process is shown in Figure 1. It initially retrieved in excess of 200,000 possible citations. We refined searches to include only full text copies available online and published after 1970 in each of the databases and in Google Scholar reducing this to 18,100 possible citations. These citations were then cross-referenced electronically with reference lists from scoping and systematic review papers published in the field of nutrition, education, and health promotion (n = 15) [15-29] published between 1997 and 2012 that yielded 454 likely studies. A final database and internet search was then conducted to identify studies published between January 2010 (year prior to publication of most recent systematic review) and May 2014. This revealed an additional 23 possible citations totalling 487 publications that were considered for review.

These 487 publications were then reviewed based on abstract and excluded if they were not conducted in primary schools or on primary school-aged children. This reduced the number of studies to 233. Studies were then excluded if they were not: a) randomised controlled trials; b) quasi-experimental studies; or c) cluster-controlled trials. This left 55 studies. On review of the full-text paper, another 6 studies were excluded for not meeting the inclusion criteria (i.e. conducted in a laboratory setting) or being a duplicate study. The final 49 studies were all in the form of peer-reviewed journal publications.

To ensure a complete review of the relevant literature is given, all 49 of the included articles are presented in Table 1. Specifically, the table outlines the details of the studies, including author(s), title, year, location, design and stated dominant theoretical framework, target population, and types of outcomes measured. The year of publication for included articles ranged from 1973 to 2011.

### Study and intervention characteristics

The final 49 studies included one randomised controlled trial, 13 quasi-experiential studies and 35 cluster-controlled trials. These studies captured data from 38001 primary school children in 13 different countries. Data capable for inclusion in the meta-analyses came from 20234 (53%) participants. All but one country (Trinidad and Tobago) included in these studies were member nations of the Organization for Economic Co-operation and Development (OECD). Only 27 of the 49 studies reported the theoretical frameworks used to inform their intervention design. Whilst some studies reported multiple theoretical approaches (see Table 1), Social Cognitive Theory was the most frequently used theoretical framework and was reported in 16 of 27 studies.

### Teaching strategies/approaches

There were eight dominant teaching strategies or approaches to teaching exhibited across the 49 studies that addressed the pre-determined areas of healthy eating for primary school students (i.e. food consumption/energy intake, fruit and vegetable consumption or preference, sugar consumption or preference, and nutritional knowledge). Some studies included more than one of these teaching strategies/approaches in their intervention group. The dominant teaching strategies/approaches were: 1) Enhanced curriculum approaches (i.e. speciality nutrition education programs beyond existing health curricula delivered by teachers or specialists) (n = 29); 2) cross-curricular approaches (i.e. nutrition education programs that were delivered across two or more traditional primary school subjects) (n = 11); 3) parental involvement (i.e. programs requiring active participation or assistance from a parent within or outside the school environment) (n = 10); 4) experiential learning approaches (i.e. school/community garden, cooking and food preparation activities) (n = 10); 5) contingent reinforcement approaches (i.e. rewards or incentives given to students in response to desired behaviours) (n = 7); 6) literary abstraction approaches (i.e. literature read by/to children whereby a character promotes/exemplifies positive behaviours) (n = 3); 7) games-based approaches (i.e. board/card games played by students at school designed to promote positive behaviour and learning of new knowledge) (n = 2); and 8) web-based approaches (i.e. internet-based resources or feedback mechanisms that could be accessed by students at home or school) (n = 2).

The results of the systematic review indicate that several dominant evidence-based approaches to teaching healthy eating in the randomised controlled trial, quasi-experimental and cluster controlled trial literature. In order to determine the strength of the evidence for these approaches, they are analysed against each of the major outcomes used to determine healthy eating and if the study achieved p-values of p < .05 for 50% of the studies, the magnitude of *M*_*d*_ (i.e. minimal, small, medium, large) and/or if *M*_*d*_ > .40. The decision to use an effect size of *M*_*d*_ > .40 is based on Hattie’s Zone of Desired Effects reside above this hinge point [11] and therefore have the greatest influence and represent the best investment for improving educational outcomes.

#### Food consumption and energy intake

Eleven studies reported on outcomes of food consumption and the overall energy intake of primary school-aged children. Curriculum-based approaches were the most popular (seven studies) and reported achieving statistical significance of p < .05 or better across nine studies reducing food consumption or energy intake outcomes. However, researchers were able to calculate effect sizes across six of the reported outcomes and found that four showed minimal or no effect, one had a negative effect and one reported a small effect size. The mean effect size of curriculum-based approaches is minimal (*M*_*d*_ = 0.12) and would suggest that curriculum-based approaches alone are not the best influence on reducing food consumption or energy intake.

Three studies utilising experiential learning approaches (i.e., school/community gardens, cooking lessons and food preparation) reported on outcomes associated with reducing food consumption and energy intake. Two of these studies reported achieving statistical significance of p < .05 or better for at least one food consumption or energy intake variable. Effect sizes could be calculated on three of the reported outcomes from two studies. Two large effect sizes were recorded and the other showed no effect. Whilst there were only a small number of effect sizes that were able to be calculated based on the reporting method in these studies, the mean effect size was *M*_*d*_ = 1.31 and within the Zone of Desired Effects. These approaches warrant greater investigation to reduce the amount of variance in the calculated effect but show promise in their ability to reduce food consumption and energy intake.

#### Fruit and vegetable (FV) consumption or preference

In terms of FV consumption or preference, curriculum-based approaches were again the most popular. 60% of curriculum-based approaches found statistically significant (p < .05) improvements in FV consumption or preference among primary school-aged children. However, it is important to note that many of the studies that used curriculum-based approaches (especially those with stronger p values) also coupled their interventions with secondary approaches (e.g., experiential-learning, parental-involvement). Given the way in which data was reported in these studies, it is difficult at this stage to determine the degree to which curriculum-based approaches alone contributed to statistical significance.

Of the 30 effect sizes that were calculated by the researchers, 33% had a medium to large effect and a further 23% had a small effect size. The mean effect size for curriculum-based approaches was *M*_*d*_ = 0.45 indicating that having a nutrition curriculum delivered in primary schools makes an important investment in improving FV consumption or preference based on the educational hinge-point of effect sizes described by Hattie [11]. All but one study that was included in the analysis appeared to be based on behavioural, mastery, or didactic approaches and curricula models. The study driven by a socio-cultural perspective of health [30] had only 33 participants and effect sizes ranging from-0.26 to 1.04 for a range of different FV consumption or preference behaviours.

Experiential-learning approaches were used in eight studies to improve FV consumption or preference in primary school children and proved to be very effective with 75% of these types of studies yielding statistical significance at p < .05 or better. Of the 11 effect sizes that were calculated by the researchers, 45% had a large effect and the remaining 55% had a minimal effect size. However, the mean effect size for experiential-learning approaches that included school/community gardens, cooking skills, or food preparation was *M*_*d*_ = 0.68, indicating experiential-learning approaches were within the Zone of Desired Effects [11] for improving FV consumption or preference in primary school children.

Cross-curricular approaches (i.e., learning experiences taught across two or more learning areas/subjects) to improving FV consumption or preference in primary school children also proved to very effective. Of the 10 studies using cross-curricular approaches, 90% of these yield statistical significance at p < .05 or better and of the 6 effect sizes calculated by the researchers, 50% had large effect sizes and the remaining 50% had a small or medium effect size. Whilst there were only a small number of effect sizes that were able to be calculated based on the reporting method in these studies, the mean effect size was *M*_*d*_ = 0.63, which was within the Zone of Desired Effects.

Four studies used a contingent reinforcement (i.e., reward for behaviour) approach in promotion of FV consumption or preference among primary school children. All four (100%) of these studies reported achieving statistical significance of at least p < .05. There were six effect sizes reported across only two studies [31,32] and four of these effect sizes (67%) were considered large and two (33%) were considered minimal. Based on these two studies, the average effect size for contingent reinforcement in promoting FV consumption or preference is *M*_*d*_ = 1.34. More studies are needed in order to ascertain an average effect size with less variance, however, based on available data this approach is well above *M*_*d*_ = 0.4 with strong statistical significance in every study indicates it is a worthwhile investment strategy in improving FV consumption or preference among primary school children.

Parental involvement was incorporated into 10 studies that reported against 23 FV consumption or preference outcomes in primary school children. 91% of the outcomes reported against were statistically significant at the p < .05 level. The researchers were able to calculate 14 effect sizes in five of the studies. The results were varied with three large, two medium, three small, two minimal and four negative effect sizes being calculated. The mean effect was *M*_*d*_ *=* 0.39 that was just below the Zone of Desired effects however it is worthwhile noting that no parent involvement approach was ever ‘stand-alone’. They all included elements of enhanced curriculum, cross-curricular, experiential learning or web-based support.

#### Sugar consumption or preference (not from whole fruit)

Enhanced curriculum approaches (mainly based on behavioural or social cognitive theories) in primary schools provided 10 studies for reducing sugar consumption or preference in students however only three yielded statistical significance of p < .05 or better for reducing any sugar-laden beverage (SLB), fruit juice or carbohydrate consumption. Six effect sizes were calculated from these studies that showed one large, one small and four minimal effect sizes. The mean effect size of curriculum approaches for reducing sugar consumption however was only *M*_*d*_ = 0.28 suggesting that greater investment beyond curriculum is required to make a substantial difference in reducing the sugar consumption of primary school children.

Cross-curricular approaches were reported in two studies [33,34] in reducing SLB or fruit juice consumption. Both studies reported statistically significant reductions in both SLB and fruit juice consumption at p < .05 or better. Taylor et al. [34] reported two minimal effect sizes whilst James et al. [35] reported a large effect size. The mean effect size for cross-curricular approaches at reducing SLB or fruit juice consumption was *M*_*d*_ = 0.42. This was within the Zone of Desired Effects [11], but more investigation may be required given the small number of studies included in the analysis.

#### Nutritional knowledge

There were 12 studies that adopted enhanced curricula approaches to improving the nutritional knowledge of primary school children. There were 13 nutritional knowledge outcomes that achieved a statistically significant improvement of p < .05 or better. In fact, 8 of the 13 studies reported statistical significance of p < .001. Researchers were able to calculate 7 effect sizes (3 × large, 1 × medium, 3 × minimal) with the mean effect size being *M*_*d*_ = 0.75. This indicates that quality curriculum interventions (largely based on behavioural or social cognitive learning theory) are capable of achieving improvements in student nutritional knowledge with the Zone of Desired Effects [11].

An experiential learning-approach was adopted in four studies and all reported achieving statistical significance of p < .05 across seven nutritional knowledge-related outcomes. The researchers were able to calculate effect sizes for six of them and found five large and one minimal effect size. The mean effect size for the experiential learning approaches to nutritional knowledge was *M*_*d*_ = 1.35 indicating this approach is a particularly strong evidence-based strategy for improving the nutritional knowledge of primary school-aged children

## Discussion

This meta-analysis of school-based teaching interventions that have focused on improving the eating habits of primary school children found that experiential learning approaches had the greatest effect on reducing the food consumption, energy intake and nutritional knowledge of primary school children, and a smaller effect on primary school children’s FV consumption or preference. The other strategies that had a smaller effect on improving primary school children’s nutritional knowledge and reducing sugar consumption or preferences were cross-curricular approaches and quality curriculum interventions, respectively. In regards with improving primary school children’s FV consumption or preferences, both cross-curricular and quality curriculum interventions were effective.

In light of these findings, it is important to note that the high levels of heterogeneity among the included primary school healthy eating programs, does not make it possible to make firm conclusions. However, the findings have been supported in other literature, with experiential learning strategies, such as garden-enhanced learning strategies, positively influencing vegetable preferences and consumption among primary school children, which has been found to be the strongest predictor of future consumption [36-39]. Similar to this review, Langellotto & Gupta [39], who used meta-analytic techniques, found that school gardens and associated teaching strategies increased vegetable consumption in children, whereas the impacts of nutrition education programs were marginal or non-significant. There are two possible reasons for these findings: 1) school gardens increase access to vegetables; and 2) gardening decreases children’s reluctance to try new foods. Birch and colleagues [38] have also stated that in order to improve primary school children’s healthy food preferences, experiences and strategies need to increase availability and accessibility to increase exposure to those foods that will then affect their willingness to taste.

Whilst some studies report FV consumption or preference independently of each other, this tends to be the exception rather than the rule of reporting FV consumption or preference in primary school-based studies. Future studies should seek to promote, analyse and report vegetable consumption independent of fruit consumption to ascertain what physiological and behavioural effects this may have on students and findings of the study. This is because excessive consumption of fruit-based sugars (i.e. consuming fructose >50 g/d) may be one of the underlying aetiologies of Metabolic Syndrome and Type 2 Diabetes [35].

This study has some important considerations with regard to its generalizability. The target population were the students attending primary schools from any country around the world but all the studies bar one [40] were conducted in nations of the OECD. As such, they represent some of the most developed and advanced economies on the planet and should be taken into serious consideration when seeking to generalise these findings. Of the 49 studies analysed, more than half (n = 28) were conducted in the United States followed by the United Kingdom (n = 7). This may be attributed of the growing percentage of children in the USA and UK with non-communicable diseases attributed to diet-related factors [4,41]. It may also be indicative of the capacity of advanced economies, such as the USA and UK, to conduct empirically robust studies in primary school settings [42].

### Strengths and limitations

There are several strengths of this systematic review and meta-analysis. First, this is the first known paper to systematically extract specific teaching strategies and approaches that facilitate the healthy eating of primary school children. As such, we conducted a systematic review using broad search terms to increase the probability of identifying all eligible publications, which yielded a well-sized (k = 49) evidence base. Second, the method of meta-analysis allowed for these strategies to be considered against other nutritional as well as the educational meta-analytic literature. Third, teaching strategies and approaches were reliably coded using schema of existing evidence of ‘what works’ in educational settings [11].

There were a few limitations associated with this review. The heterogeneity of primary school healthy eating interventions is large. This fact alone limited our ability to measure the effectiveness of each teaching strategy in the multi-faceted nutrition education programs. Moreover, it is possible that some strategies are commonly clustered with others, thus our findings should be considered carefully in terms of these strategies having similar effects when implemented on their own. Given that all the articles were identified from the peer-reviewed literature, there is some possibility of publication bias on the nature of evidence available to inform the review. Publication bias by particular journals, or more specifically the inability and discouragement of publishing articles that report negative results, may distort conclusions reached. Further, due to all but one study were conducted in OECD countries, findings from this systematic review and meta-analyses should be limited to informing decision making of stakeholders in those of similar nations.

## Conclusion

Most teaching strategies extracted from intervention studies lead to positive changes in primary school children’s nutritional knowledge and behaviours. However, the most effective strategies for facilitating healthy eating in primary school children are enhanced curricula, cross-curricula and experiential learning approaches. Other strategies that showed some promising effect, but need to be further investigated include contingent reinforcement and parental involvement approaches.

Complete citations of the studies included in the systematic review and meta analyses are listed as the following in the reference list [30-34,36,40,43-84].
